# The Rap1 Guanine Nucleotide Exchange Factor C3G Is Required for Preservation of Larval Muscle Integrity in *Drosophila melanogaster*

**DOI:** 10.1371/journal.pone.0009403

**Published:** 2010-03-03

**Authors:** Margret Shirinian, Caroline Grabbe, Milica Popovic, Gaurav Varshney, Fredrik Hugosson, Hans Bos, Holger Rehmann, Ruth H. Palmer

**Affiliations:** 1 Department of Molecular Biology, Umeå University, Umeå, Sweden; 2 Department of Physiological Chemistry, Centre for Biomedical Genetics and Cancer Genomics Centre, University Medical Center, Utrecht, The Netherlands; Stockholm University, Sweden

## Abstract

C3G is a guanine nucleotide exchange factor (GEF) and modulator of small G-protein activity, which primarily acts on members of the Rap GTPase subfamily. Via promotion of the active GTP bound conformation of target GTPases, C3G has been implicated in the regulation of multiple cellular and developmental events including proliferation, differentiation and apoptosis. The *Drosophila C3G* orthologue exhibits a domain organization similar to that of vertebrate C3G. Through deletion of the *C3G* locus, we have observed that loss of C3G causes semi-lethality, and that escaping adult flies are characterized by a reduction in lifespan and general fitness. *In situ* hybridization reveals C3G expression in the developing embryonic somatic and visceral muscles, and indeed analysis of C3G mutants suggests essential functions of C3G for normal body wall muscle development during larval stages. C3G mutants display abnormal muscle morphology and attachment, as well as failure to properly localize βPS integrins to muscle attachment sites. Moreover, we show that C3G stimulates guanine nucleotide exchange on *Drosophila* Rap GTPases *in vitro.* Taken together, we conclude that *Drosophila* C3G is a Rap1-specific GEF with important functions in maintaining muscle integrity during larval stages.

## Introduction

The ability of a cell to accurately respond to external signals in different developmental contexts relies on the integration of multiple sets of signaling pathways, which in many cases are highly conserved through evolution. A key event of such cellular signaling is the ligand-mediated activation of receptor tyrosine kinase (RTK) family proteins, leading to the recruitment of a multitude of proteins that function to transmit signals to the proper downstream targets. Essential players in this process include protein and lipid kinases, adaptor and scaffolding molecules, as well as members of the small GTPase superfamily. Small GTPases are monomeric GTP-binding proteins of 20–25kDa, which act as molecular switches during diverse cellular and developmental events, including proliferation, differentiation, apoptosis and control of the cytoskeleton [1]. The rather large superfamily of small GTPases is subdivided, based on structure and function, into the Rho, Rab, Ran, Arf/Sar and Ras (which is further divided into Ras/Ral/Rap subfamilies) GTPase families. GTPases cycle between an inactive GDP-bound and an active GTP-bound conformation, a process which is regulated by the concerted action of activating guanine nucleotide exchange factors (GEFs) and inhibiting GTPase activating proteins (GAPs) [2]. In analogy to the small GTPases, the human genome encloses a large number of selective GEF families, in which unique combinations of the GEF domain with specific protein modular domains provide activity, which is specific towards the corresponding GTPase (Bos, 2007). Similar rules also apply to vertebrate GAP proteins.

The Rap family of GTPases, and thus their associated regulators, are primarily involved in regulating cell-cell junction formation, cell adhesion to extracellular matrix and polarity [1]. One of the GEFs that has been assigned specifically to Rap1 is C3G, a multidomain protein which was originally isolated as a binding partner of the v-CRK adaptor molecule [3], interacting with the CRK SH3 domain via four proline-rich regions in the central region of the molecule [3], [4]. Later on, the C3G CDC25-Ras exchange motif (CDC25-REM) was reported to stimulate guanine nucleotide exchange on at least two Ras family members, Rap1 and R-Ras *in vitro* [5], [6].

*In vivo* studies in mouse models have shown that C3G is essential during early embryogenesis and that C3G null mutant embryos die around day 5.0 of gestation, thereby creating difficulties in the study of C3G function during development [7]. A hypomorphic allele, *C3G^gt^*, which produces less than 5% of normal C3G protein levels, allows the survival of *C3G^gt/gt^* mutants to embryonic day 14 (E14.5) [8]. *C3G^gt/gt^* mutant mice die of hemorrhaging due to vascular defects, suggesting a role for C3G in vascular myogenesis, in keeping with the lack of correctly developed supporting cells in *C3G^gt/gt^* animals. Moreover, C3G has recently been implicated in the regulation of the size of the cerebral cortex neural precursor population, and mice lacking C3G exhibit excessive proliferation in the cortical neuroepithelium [9]. Although C3G has several proposed functions in vertebrates, its physiological and biological role in *Drosophila* is unknown [10], [11], [12]. Previously, *Drosophila* C3G has been shown to have a genetic interaction with Rap1 and with components of the Ras-MAPK pathway [13], however direct evidence of *Drosophila* C3G GEF activity and specificity is lacking. In addition, the absence of *C3G* mutants has precluded an evaluation of the importance of C3G protein function in the fly.

In order to increase the understanding of C3G *in vivo*, we have generated *Drosophila C3G* null mutants and concluded that loss of C3G results in semi-lethality, with escaping adults characterized by a shorter life span and reduced general fitness. While *in situ* hybridization depicts C3G expression in embryonic CNS, somatic and visceral muscles, our analysis of *Drosophila C3G* mutant embryos has failed to reveal any major embryonic developmental defects in any of these tissues. However, when investigating the larval development of *Drosophila C3G* mutants, we detected substantial disorganization of the larval muscle architecture, as well as defects in integrin localization at muscle attachment sites. In support of a role of C3G in somatic muscles, we have also observed that misexpression of an activated form of C3G results in defective muscle structure and muscle-muscle detachment. To study the function of C3G in detail at the molecular level, we have performed guanine nucleotide exchange assays, providing conclusive evidence that *Drosophila* C3G acts as a GEF for *Drosophila* Rap1 *in vitro*. Moreover, *Drosophila* C3G also exhibits GEF activity towards human Rap1 but not H-Ras. In conclusion, we propose that C3G is an accessory component of the *Drosophila* musculature, essential for the proper localization of integrins at muscle-muscle and muscle-epidermis attachment sites and important for maintaining muscle integrity during larval stages.

## Methods

### Fly Stocks

Standard *Drosophila* husbandry procedures were applied. *Drosophila* strains were maintained on standard potato-meal medium, and raised and crossed at room temperature unless otherwise stated. The PiggyBac elements used in the study were from the Harvard Exelixis stock collection [14]. The various *UAS-C3G* transgenic flies (*UAS-C3G^WT^*, *UAS-C3G^CA^* and *UAS-C3G^DN^*) were kindly provided by Dr U. Gaul [13]. The Zasp-GFP reporter was obtained from Bloomington (stock 6338).

### Generation of *Drosophila C3G* Deletion Mutants

The *Drosophila C3G* deletion mutant (Δ*C3G^MS^*) was generated by heat shock FLP-induced FRT-mediated recombination of the transposable elements RBe03301 and XPd00064 [14]. Δ*C3G^MS^* deletion mutants were identified and verified by genomic PCR using the primers, AACAAATTGTTGTTATGCGT for the 5′ side and AGAATGCGGTGTGCCGTAAG for the 3′ side. *Drosophila Miple2* was used as a control for the genomic PCR using the primers CGAAATAAAACACTCTACC for the 5′ side and CAAATCGCCATTGGAAACTC for the 3′ side. Mutants were additionally confirmed by Southern Blot analysis.

### *Drosophila* DNA Preparation and Southern Blotting

Genomic DNA was prepared using standard techniques. Genomic DNA, digested with HindIII, EcoRI (New England Biolabs) was electrophoresed on 1% agarose gel and blotted onto Hybond N+ filter (GE Healthcare). The filter was subsequently analyzed using Digoxigenin-labeled (DIG) *Drosophila C3G* specific DNA probes, which were detected by the DIG detection chemiluminescent assay (Roche). Primers used for making the probe were CTCCATCCACGCCCGGCACCTGTT for the 5′ side and ATTGGAACTGCAGTCCAGGTCCGA for the 3′ side.

### *Drosophila* RNA Preparation and Reverse Transcription–PCR Analysis

RNA was isolated from adult *Drosophila* using RNA isolation kit (Bio-Rad). Reverse transcription was performed using the Superscript II reverse transcriptase (Invitrogen) and primers for *shifted* mRNA. The primers used were GCGGGATTTTTCAAGTGTCGAAAC for the 5′ side and CCTTTTGTCGCTGTTGCTGTTGTT for the 3′ side.

### *In situ* Hybridisation

A DIG RNA labeling kit (Roche) was applied to generate DIG-labeled RNA probes, using full-length *Drosophila C3G* cDNA (RE10624, accession number BT010019) as template. *In situ* hybridization of whole-mount wild type *Drosophila* embryos was carried out as described [15].

### Antibody Production against *Drosophila* C3G

For the preparation of *Drosophila* C3G antiserum, DNA encoding C3G amino acids 1- 155 was generated by PCR and subcloned into pETM-11. The reading frame of the His-C3G sequence was subsequently confirmed by sequence analysis. Recombinant His-C3G fusion protein was purified form *Echerichia coli* (BL21 (DE3)) bacterial lysates by standard protocols using Ni-NTA agarose (Qiagen) and subsequently used for immunization of guinea pigs.

### *Drosophila* Protein Isolation and Western Blotting

Adult *Drosophila* flies of the indicated genotypes were ground in Lysis Buffer consisting of 50 mM HEPES pH 7.4, 150 mM NaCl, 1 mM EDTA, 1 mM EGTA, 1% Triton-X-100, 10% Glycerol, 25 mM NAF, 10 µM ZnCl2 and a protease inhibitor cocktail (Complete, Roche). Lysates were cleared by centrifugation and protein concentrations determined using the Bio-Rad protein assay. Protein samples were separated on SDS-PAGE and transferred to a polyvinylidene difluoride membrane (Millipore). Membranes were blocked in 5% BSA for 1 hour, prior to incubation with either guinea pig anti-C3G, or mouse anti-α-Tubulin (Sigma) antibodies and detection by ECL (GE Healthcare).

### Lethality and Life Span Determination

For life span experiments, flies were collected during 24 hours and aged at the temperatures indicated. Flies were transferred daily onto fresh food and counted. The lethality determination was performed by placing 100 embryos of the indicated genotypes onto apple juice plates. Embryos developing into larvae and larvae enclosing into adult flies were counted.

### Immunostaining and Antibodies

Embryos were fixed and immunostained as previously described [16]. Larval muscles were heated at 60°C for 10 seconds, fixed in 4% formaldehyde, blocked in 5% NGS overnight at 4°C and stained according to standard procedures. The following primary antibodies were used: rabbit anti-dAlk (1∶1000) [17], guinea pig anti-Alk (1∶1000) [18], rabbit anti-β3-tubulin (1∶3000) [19], mouse anti-myc (1∶1000, 9E10), rabbit anti-myc (1∶250, Sigma), mouse anti-αGFP (1∶1000, Clontech), mouse anti-βPS integrin (CF.6G11, 1∶20, Developmental Studies Hybridoma Bank), Rhodamine Phalloidin, Phalloidin Alexa flour 488, Phalloidin Alexa flour 647 (Molecular Probes). The following secondary antibodies were used; biotin-conjugated anti-rabbit IgG (1∶500, Vector Laboratories), Cy3 goat anti-mouse (1∶1000, Jackson), Cy2 goat anti-mouse (1∶1000, GE Healthcare), Cy2 goat anti-rabbit (GE Healthcare), Cy3 goat anti-rabbit (GE Healthcare), Cy2 donkey anti-rat (Jackson ImmunoResearch). Embryos were cleared in methyl salicylate (Sigma) before visualization. Embryo staging was carried out according to Campos-Ortega and Hartenstein [20]. Larval muscles were mounted in Vectashield (Vector Laboratories).

### TUNEL Assay

TUNEL assay was performed according to the manufacturer's protocol (In Situ Cell Death Detection Kit, Fluorescein, Roche). Larval muscles were dissected and fixed in 4% paraformaldehyde, before being washed and permeabilized in PBS-T (0.1% Triton X-100 in PBS). Muscles were subsequently stained with the TUNEL reaction mixture for 1 hour at 37°C in the dark. Samples were rinsed three times with PBS and analyzed directly using a fluorescence microscope. As positive control permeabilized cells were incubated with DNase I (3 U ml^−1^ in 50 mM Tris-HCl, pH 7.5, 1 g l^−1^ BSA) for 10 minutes at room temperature to induce DNA strand breaks. As negative control permeabilized cells were incubated in TUNEL reaction mixture without addition of the terminal transferase.

### Recombinant Proteins for the Guanine Nucleotide Exchange Assay

Full-length *Drosophila Rap1* and *Rap2L* cDNA clones (RE42418 and RE63021, respectively) were obtained from DGRC (Drosophila Genomics Resource Center). *Drosophila C3G* RNA was isolated (Bio-Rad) and cloned by RT-PCR (Invitrogen). Fragments were cloned using BamHI/EcoRI for (C3GGEF) and EcoRI/NotI for (*Drosophila* Rap1 and Rap2L). The following constructs were generated; pGEX4T3:Rap1 (aa 1–167), pGEX4T3-1:Rap2L (aa 1-167), pGEX4T-1:C3GGEF (aa 1332–1565). Rap1B (*Homo sapiens*, aa 1–167) and H-Ras (*Homo sapiens*, aa 1–166) were expressed from ptac; Rap2B (*Homo sapiens*, aa 1–166) was expressed from pGEX4T3, C3G (*Homo sapiens*, aa 830–1078) expressed from pET15 and Sos (*Homo sapiens*, aa 564–1049) from pET28.

### Guanine Nucleotide Exchange Assay

To test the GEF activity of *Drosophila* C3G, human C3G and Sos on *Drosophila* Rap1 and Rap2, human Rap1B, human Rap2B and human H-Ras purified GTPases were preloaded with fluorescent mant-GDP (2′-/3′-O-(N'-Methylanthraniloyl)guanosine-5′-O-diphosphate) by incubating the GTPase in the presence of EDTA and an excess of mant GDP. The EDTA and free nucleotides were removed by gelfiltration [21]. Due to the high intrinsic off rate of *Drosophila* Rap2, *Drosophila* Rap2 was loaded by incubating 200nM mant-GDP with 200 nM *Drosophila* Rap2 until a stable fluorescent signal was obtained. The reaction was started by adding an excess of GDP and GEF and the fluorescence signal was measured over time (excitation wavelength is 366 nm and emission 450 nm). The buffers used are (50mM TrisHCL, pH 7.5, 500 mM NaCl (except for the Ras/Sos reaction, where buffers contained 100 mM NaCl), 5mM MgCl_2_, 5mM DTT, 5% glycerol). The reaction mixture consisted of 200nM of GTPase, 150nM RapGEF6 or 1500nM *Drosophila* C3G, human C3G or Sos, and 27 µM of GDP.

### Confocal Imaging

Confocal imaging was performed by using a Nikon confocal microscope C1, (Kangawa, Japan) fitted with Ar, He/Ne, and blue diode lasers. Digitized confocal images were assigned red and green pseudocolors for Cy3/Alexa Fluor 594 and FITC/Alexa Fluor 488 respectively. 40X and 60X objectives were used. Captured images were exported as TIFF format files and further processed using Adobe Photoshop 8.0 for figure mounting and labeling purposes. Larval muscle pictures at high magnification were taken using a Leica confocal microscope (TCS-SPE), images were assigned red (532), green (488) and Cy5 (647). At low magnification, images of larval body wall muscles were captured using an Axia Imager.Z2 system (Zeiss).

## Results

### Generation and Characterization of *C3G* Loss of Function Mutants

**Figure 1:**
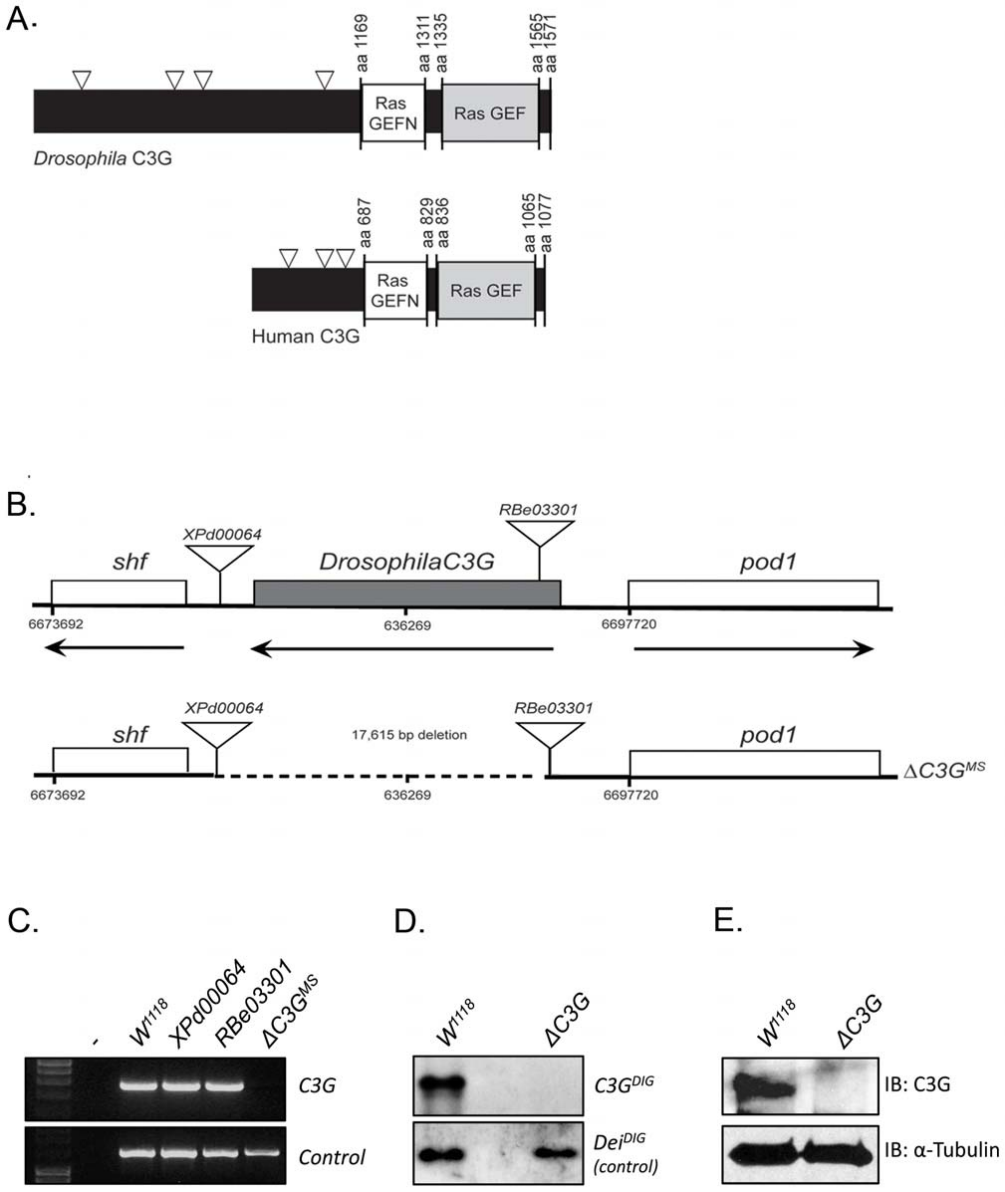
Generation of *Drosophila C3G* mutant flies. (**A**) Domain structure of *Drosophila* and human C3G. C3G consists of a long N-terminal domain (grey box), a catalytic RasGEF domain and a RasGEFN domain, which is believed to have a structural importance rather than catalytic activity; triangles indicate proline rich regions. (**B**) The *Drosophila C3G* deletion allele used in this study was generated by FLP/FRT-mediated recombination between two independent transposons (RBe03301 and XPd00064), inserted in the 5′ and the 3′ side of the *C3G* locus, respectively. This deletion results the deletion of the most of the coding region of the *C3G (ΔC3G^MS^*). (**C**) The Δ*C3G^MS^* mutant was verified by genomic PCR (lower panel) and (**D**) Southern blotting. (**E**) Western Blot analysis of the indicated genotypes confirms the absence of detectable C3G protein expression in Δ*C3G^MS^* mutants.

**Figure 2:**
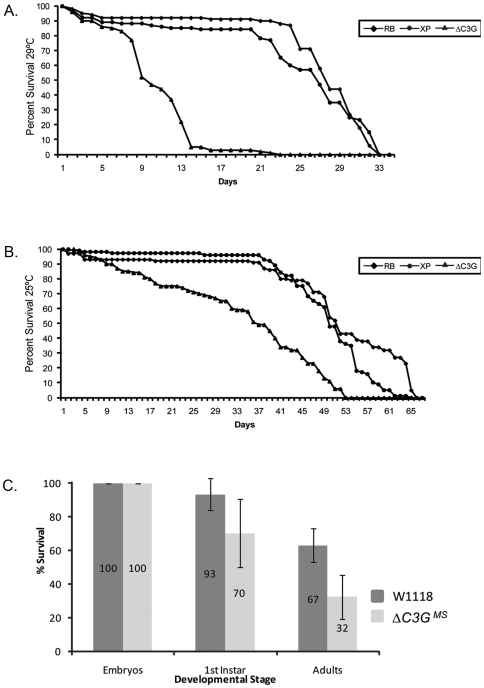
*Drosophila C3G* mutants display reduced life span and eclosion rates. (**A, B**) The life span of Δ*C3G^MS^* mutant flies was studied. Flies carrying the starting transposons (RBe03301 (RB) and XPd00064 (XP)) were analyzed in parallel, as controls. Δ*C3G^MS^* mutant flies die earlier than control flies, when aged at both 29°C (**A**) and 25°C (**B**). (**C**) Lethality determination analysis was performed on Δ*C3G^MS^* mutant and wild type animals in parallel, starting with 100 embryos of each genotype. Out of these 100, only 32% of Δ*C3G^MS^* mutants hatched into adults, which is significantly fewer when compared to 67% of controls.

Similar to its mammalian orthologues, the *Drosophila* C3G protein contains a catalytic CDC25-type GEF domain, in tandem with a conserved GEFN/REM domain, showing 67% and 39% identity to its human counterparts, respectively. Strong homology is also displayed within the N-terminal SH3 CRK binding proline-rich regions, suggesting a potential evolutionary conservation of the CRK-C3G interaction (Figure 1A, triangles) [13]. A *Drosophila C3G* loss-of-function mutant (*ΔC3G^MS^*) was generated by FLP/FRT-mediated recombination of two independent PiggyBac elements flanking the *Drosophila C3G* locus [14], which resulted in the successful deletion of most of the *C3G* coding region (Figure 1B). *ΔC3G^MS^* mutants were initially identified by genomic PCR (Figure 1C) and subsequently verified by Southern Blot (Figure 1D), inverse PCR and sequence analysis (data not shown). Accordingly, analysis of homozygous *ΔC3G^MS^* mutant flies by immunoblotting with antibodies raised against C3G showed a loss of C3G protein in the mutant animals (Figure 1E). To further confirm the specificity of the mutant we investigated the integrity of the neighboring gene on the 3′ side (*shifted*) by performing RT-PCR on mRNA extracted from *ΔC3G^MS^* flies. Indeed, *shifted* mRNA can still be detected in *ΔC3G^MS^* mutants (Figure S1). *ΔC3G^MS^* mutant flies are viable and fertile, but hatch in significantly reduced numbers, indicating semi-lethality. Analysis of the life span of *Drosophila C3G* deficient adult flies indicates a considerably shortened life span, with a survival rate of 50% after 9 days at 29°C, as compared with 26 days in controls (Figure 2A and B). A detailed examination of the viability of *ΔC3G^MS^* animals during the course of development revealed that while 70% of *ΔC3G^MS^* mutant embryos developed into larvae, only 32% eclosed into adult flies (Figure 2C). Taken together, these results suggest that *Drosophila C3G* plays a role in larval fitness and longevity of adult flies, and that *ΔC3G^MS^* animals are semi-lethal.

### *ΔC3G^MS^* Mutant Embryos Do Not Exhibit Any Obvious Embryonic Muscle Defects

**Figure 3:**
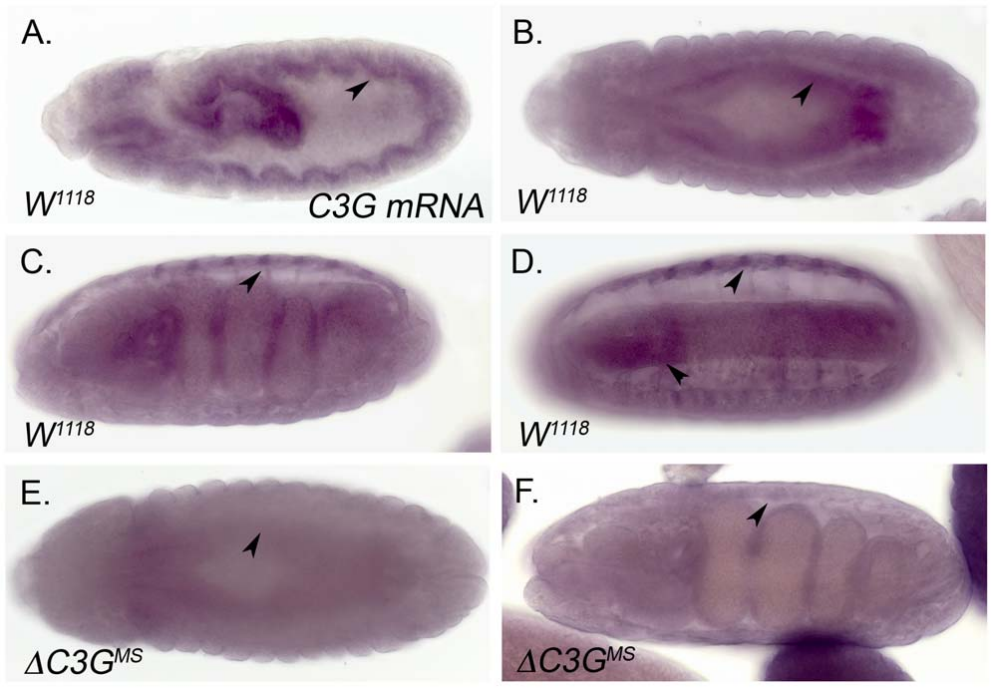
*In situ* expression pattern of *Drosophila* C3G. *In situ* hybridization using a *C3G* specific RNA probe indicates that *C3G* mRNA is expressed ubiquitously in *Drosophila* embryos, with particularly strong expression in the founder cells of the early visceral mesoderm (**A**), throughout the visceral mesoderm at later stages (**B**), as well as in somatic muscles (**C, D**) and the CNS (**D**). The absence of *C3G* mRNA could be observed in *ΔC3G^MS^* embryos (**E**, **F**).

**Figure 4:**
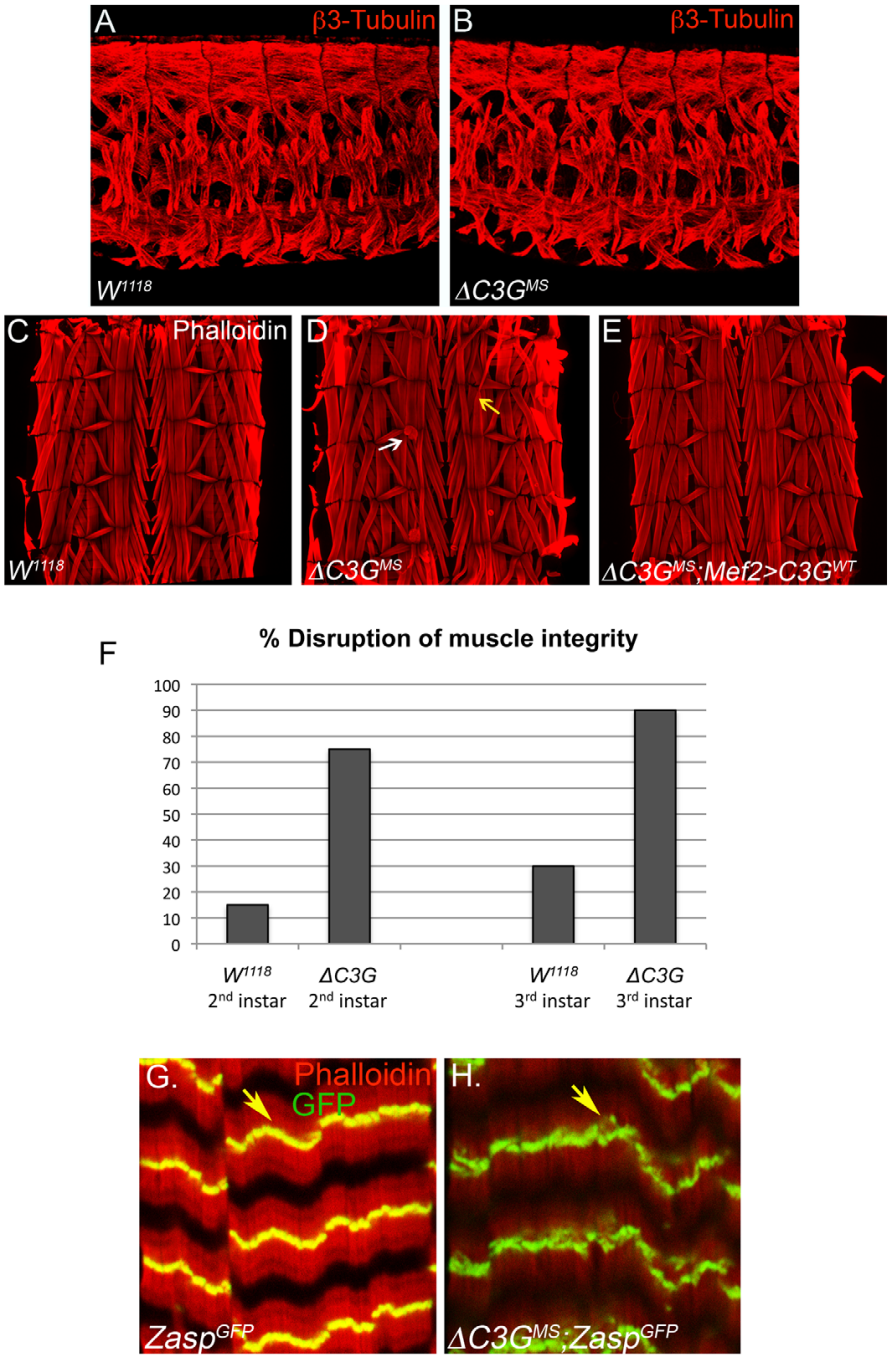
*ΔC3G^MS^* mutant animals display unique somatic muscle phenotypes during larval development. Wild type and ***Δ*** *C3G^MS^* mutant embryos were stained with anti-β3-tubulin antibodies (red) to visualize somatic muscles. (**A, B**) Similar to wild types, Δ*C3G^MS^* mutants develop a normal somatic musculature at the end of embryogenesis. (**C, D**) Third instar Δ*C3G^MS^* larvae exhibit morphological defects in the muscles (including muscle mistargeting and detachment as well as muscle “thinning”, specifically in the ventral longitudinal muscles (**D**; arrows). (**E**) The somatic muscle defects observed in Δ*C3G^MS^* mutant larvae can be rescued by GAL4/UAS-mediated (*ΔC3G;Mef2-GAL4>UAS-C3G^WT^*) reintroduction of wild type C3G in muscles (**E**). (**F**) Quantification of larval muscle phenotypes at the 2^nd^ and 3^rd^ instar larval stages, respectively. (**G, H**) Investigation of Z-band structures in wild type and ***Δ*** *C3G^MS^* mutants, utilizing a reporter for the Z-band specific protein Zasp, Zasp-GFP. Defects in Zasp-GFP distribution can be observed in Δ*C3G^M S^* muscles (**H**; arrows).

During embryonic stages *Drosophila C3G* is expressed in the visceral and somatic mesoderm as well as the CNS (Figure 3 and [13]), suggesting a role for C3G during the development of these tissues. In agreement with previous reports [13] we observe that *C3G* is expressed in the founder cells of the developing visceral mesoderm (Figure 3A). Founder cells are essential for the formation of the midgut musculature, and indeed flies lacking these cells - such as mutants for the RTK Alk and its ligand Jeb - do not form a functional midgut [17], [18], [22]. However, investigation of the visceral muscle fusion process in *ΔC3G^MS^* mutant animals indicates that C3G activity is not essential for this process (Figure S2 C and D). In addition, the analysis of the embryonic somatic musculature of *ΔC3G^MS^* mutant embryos, employing anti-β3-tubulin antibodies, failed to reveal any obvious defects also in this muscle type (Figure 4A and B). Further, the localization of βPS integrins at muscle attachment sites did not display any obvious abnormalities in *ΔC3G^MS^* mutant embryos ( Figure S2 A and B). Thus, both the visceral and somatic muscles appear to be grossly normal in *ΔC3G^MS^* mutant embryos and we were unable to identify any major defects in embryonic muscle morphology, attachment or migration (Figure 4A, B and Figure S2). Importantly, when analyzing *ΔC3G^MS^* mutants at later stages, we could conclude that the morphology of larval and adult guts was also generally normal (data not shown). Taken together, this data indicates that formation of the gut musculature proceeds normally in *ΔC3G^MS^* mutant animals, and by extension that *Drosophila* C3G is not required for gross visceral muscle development.

### *ΔC3G^MS^* Mutant Larvae Display a Distinct Somatic Muscle Phenotype

Given the absence of embryonic phenotypes, we continued our studies by examining the larval musculature in Δ*C3G^MS^* mutants. To do this, body wall muscles from third instar larvae were stained with phalloidin to visualize the actin cytoskeleton, together with βPS integrin, marking muscle attachment sites. A significant proportion (90%, n = 30) of Δ*C3G^MS^* mutant specimens analyzed showed abnormal muscle architecture of the ventral longitudinal muscles. These experiments were repeated in a double-blind manner with similar results. The Δ*C3G^MS^* mutants most commonly displayed phenotypes including deformations and detachment of the ventral longitudinal muscles (compare Figure 4D with wild type in 4C, arrows in 4D indicate muscle detachment, mistargeting and “muscle thinning”), which was observed in roughly 90% of the muscle samples analyzed (Figure 4F). While muscle defects were not observed in late embryonic stages, we were able to clearly observe defects in dissected second instar Δ*C3G^MS^* mutant larva, suggesting that the phenotype arises after embryogenesis (Figure S3). In addition, a disorganization of the sarcomeric muscle architecture in Δ*C3G^MS^* mutant animals can be observed using the Z-band specific reporter Zasp-GFP [23] (Figure 4G and H; arrow). The muscle defects observed were rescued by the reintroduction of wild type C3G protein expression (*UAS-C3G^WT^*) under control of the muscle specific Mef2-Gal4 driver in Δ*C3G^MS^* mutants (75% rescue; n = 34) (Figure 4E and data not shown).

**Figure 5:**
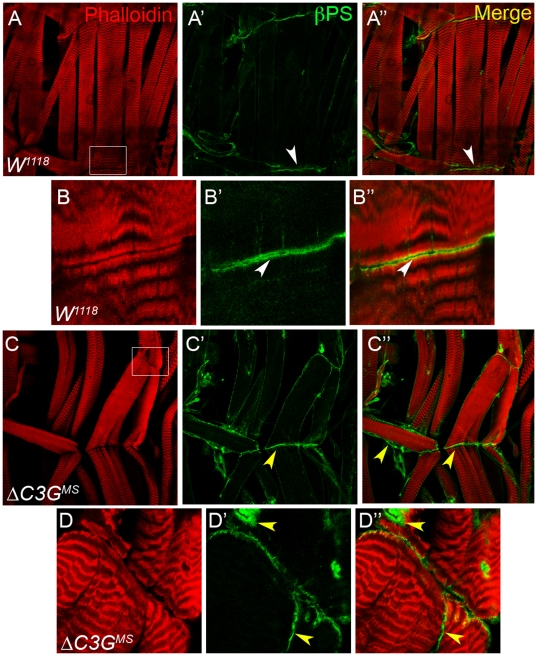
Localization of the βPS integrin at the muscle attachment sites is affected in Δ*C3G^MS^* mutant larvae. Third instar wild type control (**A, B**) and Δ*C3G^MS^* mutant (**C, D**) larval muscles were dissected and stained with Phalloidin to visualize the muscles (red) and anti-βPS integrin antibodies to depict muscle attachment sites (green). In contrast to wild type animals, the localization of βPS integrins was not restricted to the attachment sites in Δ*C3G^MS^* mutant muscles (yellow arrowheads). Instead of being concentrated at the attachment between two adjacent ventral longitudinal muscles (**C′**, **D′**), βPS was also visible around the ventral longitudinal muscle membrane (compare to **A′**, **C′**).

Given the clear defects in the larval musculature of Δ*C3G^MS^* animals, we investigated whether any of the molecules known to be important for muscle development in *Drosophila* was affected in the mutants. Examination of βPS integrin, which is normally localized at muscle attachment sites, revealed an irregular pattern in Δ*C3G^MS^* mutants (Figure 5). Whereas βPS integrin was indeed found at attachment sites in Δ*C3G^MS^* mutants, a significant amount could also be detected throughout the muscle fibers, rather than being concentrated exclusively at the muscle-muscle and muscle-epidermis attachment sites. (Figure 5C and D′′). These results suggest that although C3G is not critical for embryonic myogenesis, it appears to be required for the maintenance of muscle morphology and proper attachment during larval stages of muscle development. Notably, this is in agreement with the observation that 68% of Δ*C3G^MS^* mutant larvae fail to hatch into adult flies, which in part may be due to abnormal muscle fiber development.

Morphological defects, similar to those displayed by Δ*C3G^MS^* mutant muscles, have also been reported for mutants in genes with established roles in the maintenance of muscle integrity as well as prevention of muscle degeneration and apoptosis, such as the Dystrophin family of proteins [24], [25]. To investigate whether *Drosophila* C3G functions in similar pathways, we wished to examine whether Δ*C3G^MS^* mutant larval muscles exhibit increased levels of apoptosis. However, when performing TUNEL assays to detect DNA fragmentation as readout of apoptosis, we were unable to detect any significant difference, when comparing Δ*C3G^MS^* to wild type (Figure S4).

### Overexpression of Activated C3G Potently Induces Muscle Phenotypes

**Figure 6:**
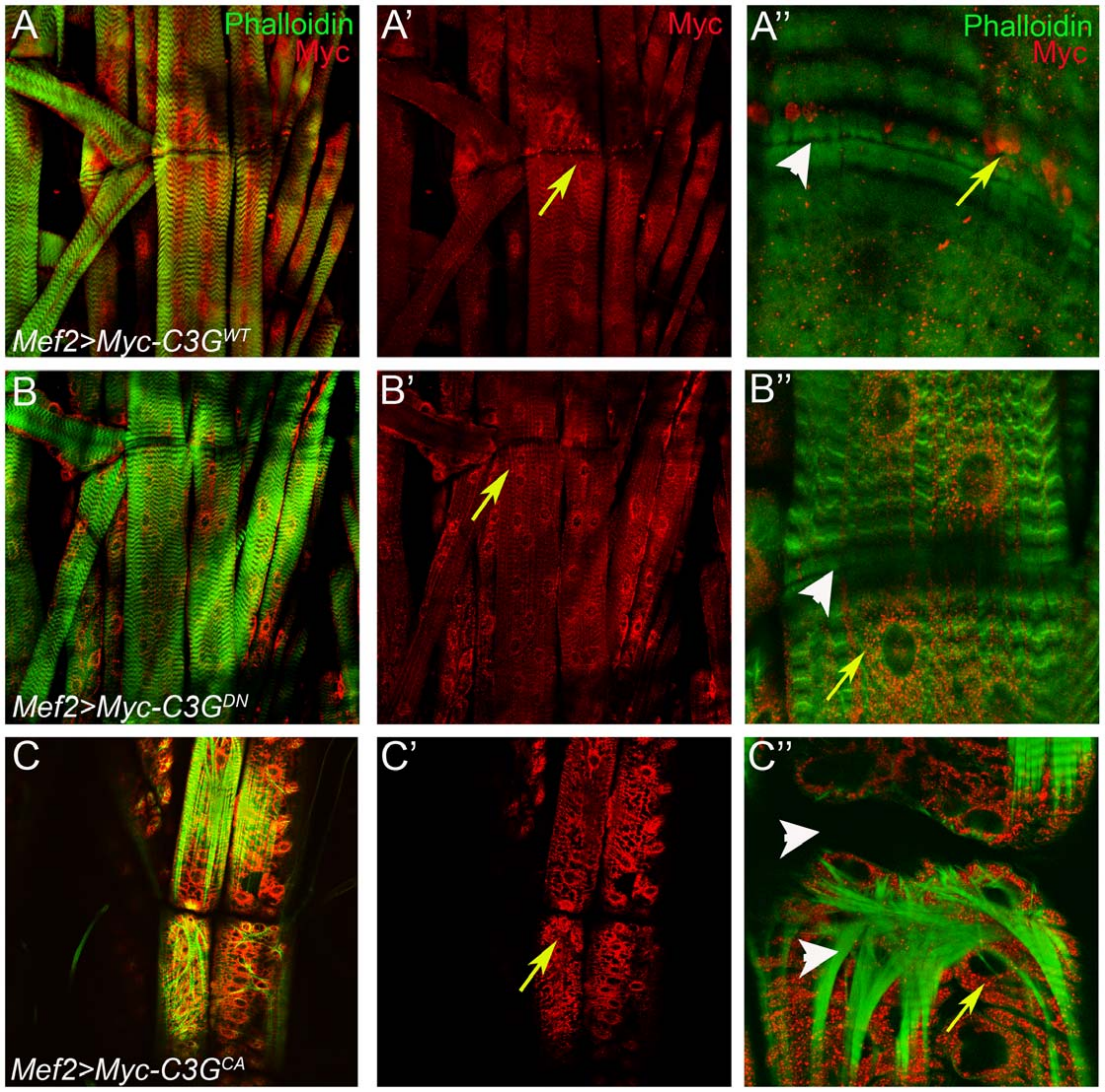
Overexpression of *Drosophila* C3G in larval muscles. Wild type (C3G^WT^), dominant negative (C3G^DN^) and activated (C3G^CA^) myc-tagged variants of C3G were overexpressed in larval muscles using the muscle specific Mef2-Gal4 driver. Third instar larval muscles were dissected and stained with phalloidin (green) to visualize muscles and anti-myc antibodies to detect C3G (red). (**A-A″**) Mef2-GAL4 induced C3G^WT^ protein localizes around the muscle nuclei and additionally accumulates at one side of the attachment site between two ventral longitudinal muscles; arrows in (**A′, A″**). (**B-B″**) Muscles overexpressing C3G^DN^ mainly show a perinuclear localization of C3G^DN^, resembling the pattern of C3G^WT^; arrow in (**B′**). (**C-C″**) Muscles overexpressing the activated form of C3G (C3G^CA^); arrows (**C′, C″**) indicate localization of also C3G^CA^ around the nuclei in the muscles. Muscle fibers appear disorganized. Arrowheads in (**C″**) indicate VL (Ventral Longitudinal) muscle detachment and the defects in actin filament structure.

**Figure 7:**
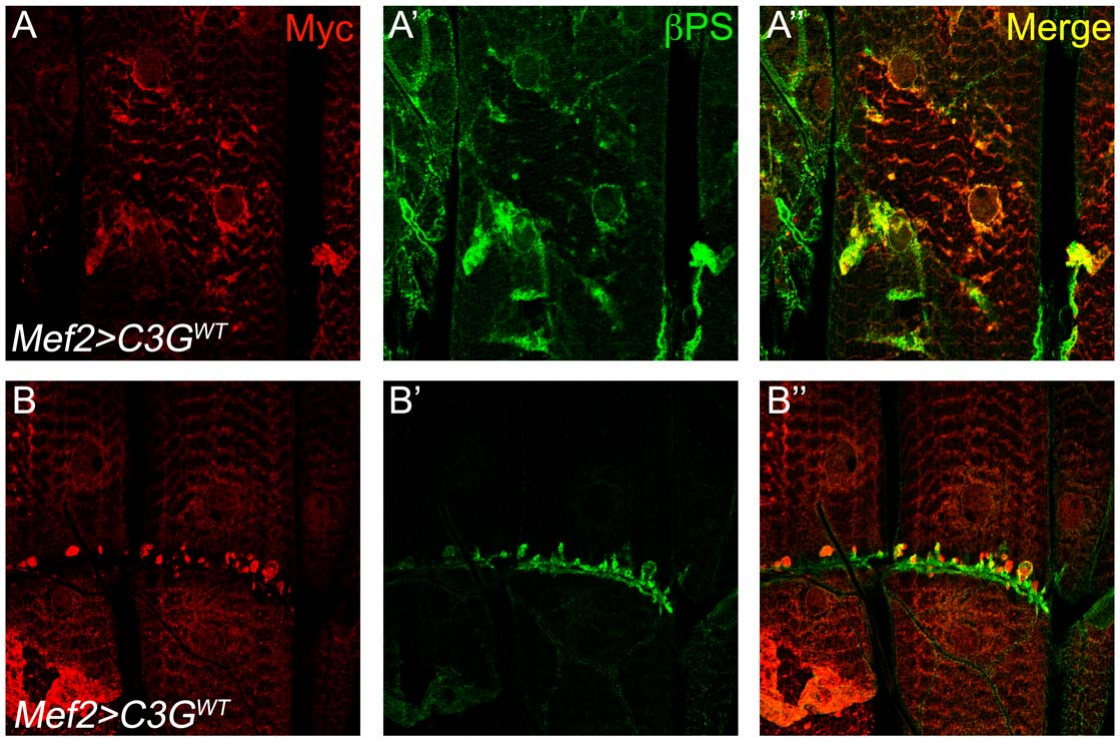
βPS integrins are recruited to sites of misexpressed C3G^WT^ protein. (**A–B**) Mef2-GAL4 was used to drive expression of wild type (C3G^WT^) specifically in muscles. Body wall muscles from 3^rd^ instar larvae were dissected and stained with antibodies recognizing Myc-C3G^WT^ (red) and βPS (green), respectively. βPS was observed to partially co-localize with C3G^WT^, both in perinuclear regions (**A–A″**) and in the accumulations of C3G^WT^ protein at one side of muscle attachment sites (**B–B″**).

In order to better understand the function of C3G during muscle development, we examined the effect of C3G gain-of-function in larval muscles. To do this we utilized the UAS/GAL4 system to specifically express transgenic constructs encoding either wild type, dominant negative (a truncated version of C3G lacking the C-terminal CDC25 catalytic domain) or constitutively activated C3G (a membrane-targeted version of C3G) in somatic muscles [13]. The different C3G variants displayed distinct patterns of subcellular localization, as well as effects on muscle morphology. Overexpression of wild type C3G (Mef2-Gal4>UAS-C3G^WT^) resulted in an intriguing accumulation of C3G both in a punctuate manner and on one side of the muscle attachment site (Figure 6A; yellow arrows). The dominant negative C3G (Mef2-Gal4>UAS-C3G^DN^) was localized in a punctuate pattern, spread throughout the muscle fibers with accumulation in perinuclear regions (Figure 6B; yellow arrows). Mef2-Gal4>UAS-C3G^DN^ flies were viable and showed no obvious alterations in actin organization (Figure 6B). The strongest effect was undoubtedly observed upon overexpression of activated C3G (Mef2-Gal4>UAS-C3G^CA^), which when expressed at 25°C resulted in embryonic lethality. Examination of somatic muscles of Mef2-Gal4>UAS-C3G^CA^ animals at embryonic stages revealed severe muscle defects (Figure S5), in contrast to embryos expressing wild-type or dominant negative C3G which were indistinguishable from controls. Expression of UAS-C3G^CA^ at 18°C allowed development of larvae displaying severe morphological defects and disruption of actin organization in somatic muscle (Figure 6C). Detachment of the ventral longitudinal muscles was also frequently observed (Figure 6C″; arrowhead). Notably, in all cases C3G was found to decorate the muscles in a punctuate pattern, as well as around the muscle nuclei (arrows Figure 6B″, C″). Given the mislocalization of βPS-integrin in Δ*C3G^MS^* mutant muscles we decided to investigate the effect of C3G overexpression on βPS-integrins. Overexpression of wild type C3G (Mef2-Gal4>UAS-C3G^WT^) resulted in a clear accumulation of β-integrin together with C3G (Figure 7), suggesting that C3G may play a role in regulation of βPS-integrin localisation in muscles.

### C3G Shows Specific GEF Activity towards *Drosophila* Rap1 but Not Rap2 *In Vitro*

**Figure 8:**
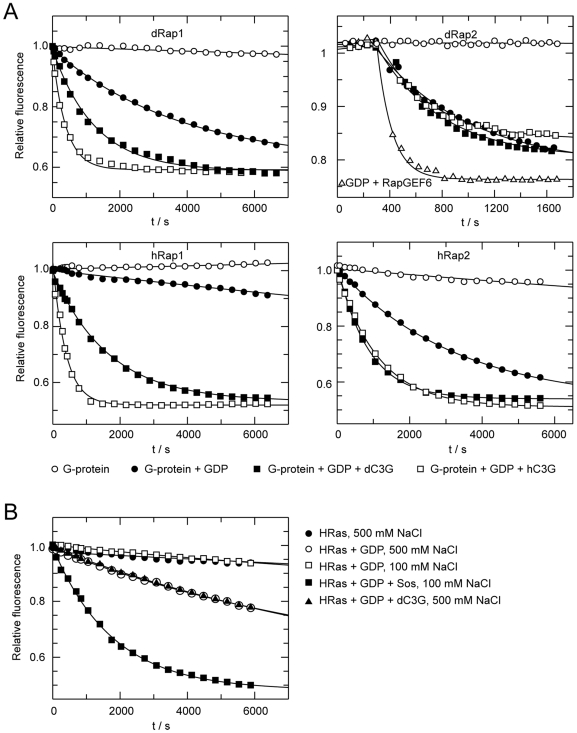
Guanine nucleotide exchange rates of *Drosophila* and human Rap proteins are stimulated by C3G. (**A, B**) The guanine nucleotide exchange activity of *Drosophila* and human C3G were analysed *in vitro*. Briefly, GTPases were preloaded with the fluorescent GDP analogue mant-GDP. The fluorescence intensity of mant-GDP is approximately double that bound to the GTPase as free in solution. In the presence of an excess unlabelled normal GDP mant-GDP is exchanged for GDP, causing a decay of the fluorescence signal, allowing a direct measurement of the exchange rate. Given that the protein is stable, a constant fluorescence signal is expected in the absence of GDP. Addition of GDP monitors the intrinsic exchange rate of the GTPase, which should be enhanced in the presence of a GEF. (**A**) The exchange rates of *Drosophila* (upper panel) or human (lower panel) Rap1 and Rap2 were analysed in the presence or absence of either the *Drosophila* or human C3G RasGEF domain. Both *Drosophila* and human C3G stimulated nucleotide exchange from *Drosophila* Rap1, whereas *Drosophila* C3G did not exhibit activity towards *Drosophila* Rap2. RapGEF6 was employed as a positive control for GEF activity towards *Drosophila* Rap2. *Drosophila* C3G stimulated nucleotide release from both human Rap1 and less efficiently from human Rap2. (**B**) In contrast to human Sos, *Drosophila* C3G was unable to function as a GEF for H-Ras, confirming the function of *Drosophila* C3G as a Rap-GEF.

*Drosophila* contains two Rap proteins, both of which are putative targets for the GEF activities of C3G. To test if *Drosophila* C3G functions as a GEF for the *Drosophila* small GTPases Rap1 and Rap2, we performed *in vitro* GDP release assays, using the RasGEF domain of C3G together with Rap1 or Rap2. These assays establish that *Drosophila* C3G stimulates guanine nucleotide release from *Drosophila* Rap1, but not Rap2 (Figure 8A). Since *Drosophila* and human C3G are closely related and share a high homology in their catalytic domains, we also examined whether human C3G acts as a GEF for *Drosophila* Rap1 and Rap2. Similar to the *Drosophila* C3G, human C3G indeed stimulated guanine nucleotide release from *Drosophila* Rap1 but not Rap2 (Figure 8A). To demonstrate the general ability of our assay to identify GEF activity towards Rap2, RapGEF6 (PDZ-GEF2) was included as a positive control (Figure 8A). The reciprocal experiment was subsequently carried out to test whether *Drosophila* C3G shows any GEF activity towards human Rap1 (hRap1B) and Rap2 (hRap2B) (Figure 8A). *Drosophila* C3G stimulates guanine nucleotide release from human Rap1 as well as Rap2, but activity towards Rap2 is less efficient than towards Rap1. Finally, we examined the GEF activity of *Drosophila* C3G towards H-Ras and could conclude that C3G does not stimulate efficient nucleotide exchange from H-Ras, in contrast with the established Ras-GEF Sos, which was employed as positive control (Figure 8B). Thus, at least *in vitro*, *Drosophila* C3G appears to act as a Rap1 specific GEF.

## Discussion

In this paper we report that loss of the guanine nucleotide exchange factor C3G in *Drosophila* causes semi-lethality, and that C3G null mutants (Δ*C3G^MS^*) display defects in larval muscle architecture and decreased survival during both larval and adult phases. Both the muscle defects and lethality observed in Δ*C3G^MS^* animals can be significantly rescued by the ectopic expression of wild type C3G protein in muscles with the Mef2-Gal4 driver, suggesting that the C3G GEF protein performs important functions in maintaining somatic muscle integrity. Our detailed analysis of the Δ*C3G^MS^* mutants resulted in several important findings. Firstly, *Drosophila* C3G is crucial for the formation of normal larval body wall muscle structure, in particular the morphology and attachment of the ventral longitudinal muscles. During larval movement, it is the contraction of these longitudinal muscles, which alternately shorten the length of these segments uni- and bilaterally, that drives locomotion. The deformed morphology of these muscles may be the underlying explanation for the requirement of C3G for larval fitness. One possibility is that the altered integrin localization in Δ*C3G^MS^* mutants arises as a result of distorted muscle activity due to aberrant sarcomeric muscle structure. Indeed, in both *Drosophila melanogaster* and *Caenorhabditis elegans* integrins are required for proper sarcomere assembly and Z band formation [26], [27], [28], [29]. In *Drosophila* absence of integrin function causes muscle detachment with no defects in the initial specification and fusion of muscle cells [26]. PS integrin deficiency has in cell culture systems been linked to loss of sarcomeric structures [27], thus suggesting a role for integrins in linking muscle attachment sites with the actin-myosin contractile apparatus [27], [30], [31], [32]. In agreement, C3G deficient mice fibroblasts display cell adhesion defects and lack paxillin and β1-integrin positive cell adhesions [8]. The panel of proteins involved in linking attachments and the sarcomeric muscle structures is constantly expanding. One such protein is the actin-binding protein α-actinin. *Drosophila α-actinin* mutant flies exhibit flight muscle paralysis [33], and indeed, upon close inspection using electron microscopy, mutant muscles show irregularities and dissolution of Z-bands, indicating a role for α*-actinin* in anchoring and stabilizing the thin filaments of the muscles. Furthermore, a recent study in *Drosophila* has shown the PDZ-LIM domain containing protein Zasp to interact genetically with integrins, which is interesting given that *zasp* mutants do not form Z-bands and fail to recruit α*-actinin* to the Z-band, resulting in muscle detachment at the onset of contractility [23]. Whereas Zasp localizes correctly at the Z-band in *ΔC3G^MS^* mutants, the Z-band itself is irregular, raising the possibility that the integrin mislocalization observed in *ΔC3G^MS^* mutant animals may partially be the result of defective anchorage of actin filaments to the Z bands. Other LIM domain adaptor proteins such as Paxillin and PINCH have also been proposed to strengthen integrin linkage to the actin cytoskeleton [34], [35], but whether the localization of these molecules is affected in Δ*C3G^MS^* mutants has not been analyzed.

In mammalian cells, endogenous C3G has been shown to interact with E-cadherin, a key component of cell-cell junctions, a binding that in the yeast-two-hybrid system was further mapped to the intracellular tail of E-cadherin [36]. The interaction between C3G and E-cadherin is induced upon cell-cell junction disassembly and is temporally linked to the activation of Rap1, which has been proposed to occur at Rab11-positive recycling endosomes [37]. Based on these findings, the internalization and disassembly of E-cadherin adhesive complexes may at a given point along the endocytic route expose the E-cadherin C3G binding site, enabling activation of Rap1, which appears to be a key determinant of E-cadherin recycling and formation of new cell-cell junction complexes [36], [37]. Besides E-Cadherin, C3G-mediated activation of Rap1 has been reported to occur also in response to other stimuli, including Nectin-mediated adhesion and mechanical stress. Nectins are immunoglobulin-like transmembrane proteins that can form homophilic or heterophilic complexes at the adherence junctions [38], supporting a role of *Drosophila* C3G at sites of cell-cell contact. The C3G-induced activation of Rap1 in response to the mechanical stress emanating from cell stretching [39] has shown to be recognized by the p130Cas adaptor protein, which in turn recruits CRK, a constitutive binding partner of C3G. Taken together, this clearly demonstrates that C3G functions as an activator of Rap1 and that this activation is involved in the regulation of cell adhesion. In agreement with this, Ishimaru and coworkers have reported that reduction of the gene dosage of Rap1 or components of the Ras-MAPK pathway can reduce the phenotypes induced by ectopic C3G overexpression [13]. Muscle attachment sites are enriched with transmembrane proteins (primarily integrins), which are required for the initial establishment of attachments, as well as a multitude of proteins that function to stabilize the attachments by linking integrins to actin filaments, including Talin and α-actinin [23], [33], [34], [35], [40]. Our data from the analysis of Δ*C3G^MS^* mutants, together with C3G overexpression studies, suggests that C3G acts as one such stabilizing protein required for preserving muscle integrity.

Our nucleotide exchange activity assays revealed that *Drosophila* C3G specifically exerts GEF activity towards *Drosophila* Rap1, but not markedly towards Rap2. Furthermore, both *Drosophila* and human C3G display an ability to stimulate nucleotide exchange of both human Rap1 and, to a lesser extent, human Rap2B. Human C3G was previously shown not to act on human Rap2A [41], which implicates a conserved tendency of C3G to act more efficiently on Rap1 small GTPases than the Rap2 subfamily. This is the first *in vitro* evidence showing that *Drosophila* C3G selectively functions as a GEF for Rap1. The *Drosophila* genome contains one annotated Rap1 protein (*roughened*) and one Rap2 family member (*rap2L*) [42]. Besides C3G, the *Drosophila* PDZ-GEF (*gef26*) has also been reported as a GEF for *Drosophila* Rap1, and has been implicated *in vivo* in photoreceptor development as well as the regulation of cell migration and morphogenesis in wing disc epithelia, dorsal closure and hemocyte migration [43], [44], [45]. However, it has not been investigated whether Rap1 has any role in the development of the larval musculature. *Drosophila* Rap2L has not been extensively studied *in vivo* and no mutants have been described at this point. Furthermore, it is not known which GEF proteins are responsible for the activation of *Drosophila* Rap2L, although our results indicate that it is unlikely to be activated directly by *Drosophila* C3G. In the GEF assays performed here we noted a high intrinsic exchange activity of *Drosophila* Rap2L, a feature which potentially could be explained by the slight sequence difference in the GTP-binding (NKXD) motif displayed by *Drosophila* Rap2L, as compared to the other *Drosophila* and human Rap proteins as well as most other Ras proteins.

The identification here of a function for C3G in the somatic musculature adds another GEF to the list of GTPase modulators with described roles in muscle development. Previousely identified GEFs include Myoblast city (MBC) which functions as a GEF for Rac1, playing a critical role in the myoblast fusion process [46], [47]. Additionally, the ARF6 GEF Loner also displays myoblast fusion defects [48]. To these we now add a role for the Rap GEF C3G in the regulation of the integrity of the larval musculature.

The punctuate and perinuclear localization of C3G upon overexpression is intriguing, although this pattern and its relevance for muscle integrity and attachment is unclear. In addition to their sarcomeric architecture, muscles contain tubular plasma membrane invaginations (transverse tubules or T-tubules) that are important for the connection between neuronal depolarization and muscle contraction. A number of proteins have been reported as critical for the biogenesis of these tubular structures. Two of these are Amphiphysin and Caveolin, which both have established functions in membrane dynamics [49], [50], [51], [52]. It is conceivable that C3G is also required for the normal biogenesis of T-tubules and if so, that the pattern of misexpressed activated C3G in muscles is the result of defective T-tubule formation. However, there is also a possibility that the punctuate pattern observed upon overexpression of wild type C3G may be related to integrin trafficking events at the attachment sites. A number of recent studies in cell culture systems have provided insights into how integrin trafficking is regulated by the Rab family of small GTPases [53], [54], and it has additionally been shown that vesicular import of integrins is an essential event during cytokinesis. It is therefore imaginable that such a mechanism of integrin regulation also exists in *Drosophila* muscles.

Taken together, the data we have presented here provide the first *in vivo* evidence for a requirement of the guanine nucleotide exchange factor C3G in the preservation of muscle integrity during larval development. Further studies are clearly needed to fully understand the function of C3G, explain the phenotypes observed in C3G deficient animals, and uncover the molecular machinery that together with C3G regulate muscle attachment, integrity, contractility and muscle maintenance.

## Supporting Information

Figure S1 *shifted* is expressed in Δ*C3G^MS^* mutants. RT-Reverse transcription PCR was performed on RNA extracts from wild type flies, flies carrying the starting transposable elements (RBe03301 and XPd00064) and Δ*C3G^MS^* mutants. *shf (shifted)* is expressed in Δ*C3G^MS^* mutants.
(0.96 MB TIF)

Figure S2 Δ*C3G^MS^* mutant embryos undergo normal fusion and migration of somatic and visceral muscles. To visualize the somatic muscle attachment sites, wild type and Δ*C3G^MS^* mutant embryos were stained with βPS integrin (red) and β3-tubulin (green) antibodies. βPS integrin localization appears to be normal in Δ*C3G^MS^* mutant embryos (A, B). (C, D) Wild type and Δ*C3G^MS^* mutant embryos were stained with anti-Alk antibodies (green) to visualize the visceral muscles and anti-Connectin antibodies (red) as a cytoskeletal marker in the visceral mesoderm. The founder cells and fusion competent myoblasts fuse normally in Δ*C3G^MS^* mutant embryos (D).
(4.02 MB TIF)

Figure S3 Δ*C3G^MS^* mutant larvae display muscle abnormalities already at the 2^nd^ instar stage of development. (A, B) Wild type and Δ*C3G^MS^* mutant larvae were dissected at the 2^nd^ instar stage and stained with Phalloidin to visualize the body wall musculature. Whereas wild type animals (A) at this stage display regular and robust longitudinal muscle fibers, the Δ*C3G^MS^* mutants (B) are characterized by thin longitudinal muscle fibers that tend to be both mistargeted and in some cases detached (right panel).
(2.22 MB TIF)

Figure S4 Δ*C3G^MS^* mutant muscles do not display increased apoptosis. Third instar wild type and Δ*C3G^MS^* mutant larval muscles were dissected and subjected to a TUNEL assay. DNA fragmentation, characteristic for apoptosis, was analyzed by fluorescent labeling (Green). Samples treated with DNAse I were used as positive controls (A'). As negative control samples were treated with the TUNEL reaction mixture, but without addition of the terminal transferase. No apoptosis was observed in Δ*C3G^MS^* mutant larva, similar to wild type muscles (D'-C').
(7.43 MB TIF)

Figure S5 Embryonic muscle development is affected by C3G ^CA^, but not C3G ^WT^ or C3G ^DN^ misexpression. (A–D) Mef2-GAL4 was used to drive expression of wild type (C3G ^WT^), dominant negative (C3G ^DN^) or activated (C3G ^CA^) specifically in muscles. Late stages embryos were collected and stained with anti-β3-tubulin (green) and anti-βPS (red) antibodies. The integrity of the embryonic somatic musculature and the targeting of integrins to muscle attachment sites was not affected by misexpression of either C3G ^WT^ (B) or C3G ^DN^ (C). However, misexpression of C3G ^CA^ resulted in muscle defects of variable magnitudes (D).
(4.03 MB TIF)
