# Correction: The Rap1 Guanine Nucleotide Exchange Factor C3G Is Required for Preservation of Larval Muscle Integrity in *Drosophila melanogaster*

**DOI:** 10.1371/annotation/c3ebafa0-1531-4e9f-ab3a-660db7b8dcd2

**Published:** 2010-03-30

**Authors:** Margret Shirinian, Milica Popovic, Caroline Grabbe, Gaurav Varshney, Fredrik Hugosson, Hans Bos, Holger Rehmann, Ruth H. Palmer

The author order of the published article was incorrect. The correct order is: Margret Shirinian, Milica Popovic, Caroline Grabbe, Gaurav Varshney, Fredrik Hugosson, Hans Bos, Holger Rehmann, Ruth H. Palmer. Milica Popovic and Caroline Grabbe should be considered equally contributing second authors. The citation now reads: Shirinian M, Popovic M, Grabbe C, Varshney G, Hugosson F, et al. (2010) The Rap1 Guanine Nucleotide Exchange Factor C3G Is Required for Preservation of Larval Muscle Integrity in Drosophila melanogaster. PLoS ONE 5(3): e9403. doi:10.1371/journal.pone.0009403. The affiliations do not change.

