# Supplementary figures and images for: The Rap1 Guanine Nucleotide Exchange Factor C3G Is Required for Preservation of Larval Muscle Integrity in Drosophila melanogaster
Source: PLoS One. 2010 Mar 3;5(3):e9403. doi: 10.1371/journal.pone.0009403 (PMC2831063; doi:10.1371/journal.pone.0009403)

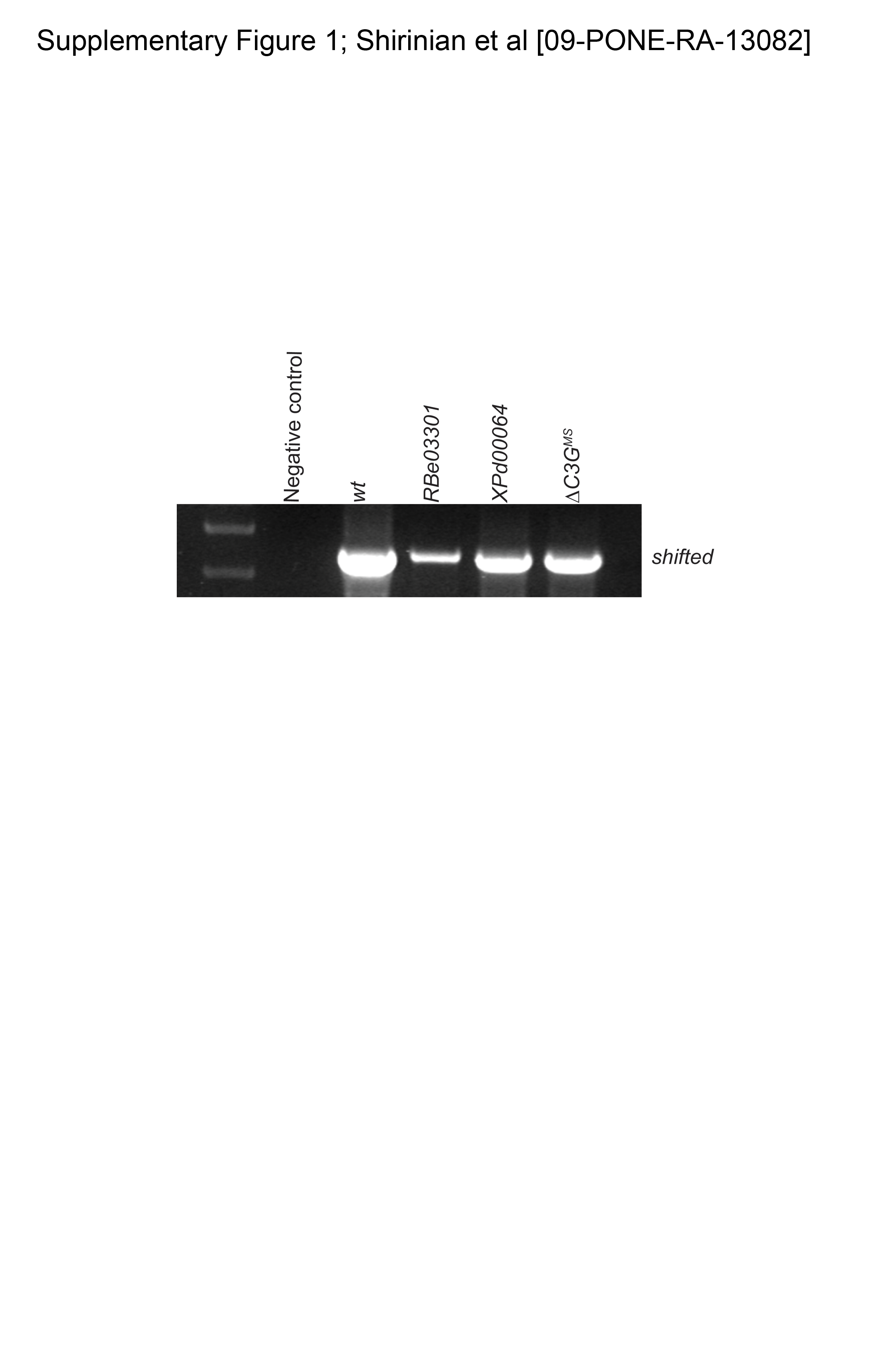

Supplement: Figure S1 — shifted is expressed in ΔC3GMS mutants. RT-Reverse transcription PCR was performed on RNA extracts from wild type flies, flies carrying the starting transposable elements (RBe03301 and XPd00064) and ΔC3GMS mutants. shf (shifted) is expressed in ΔC3GMS mutants. (0.96 MB TIF) [file pone.0009403.s001.tif]

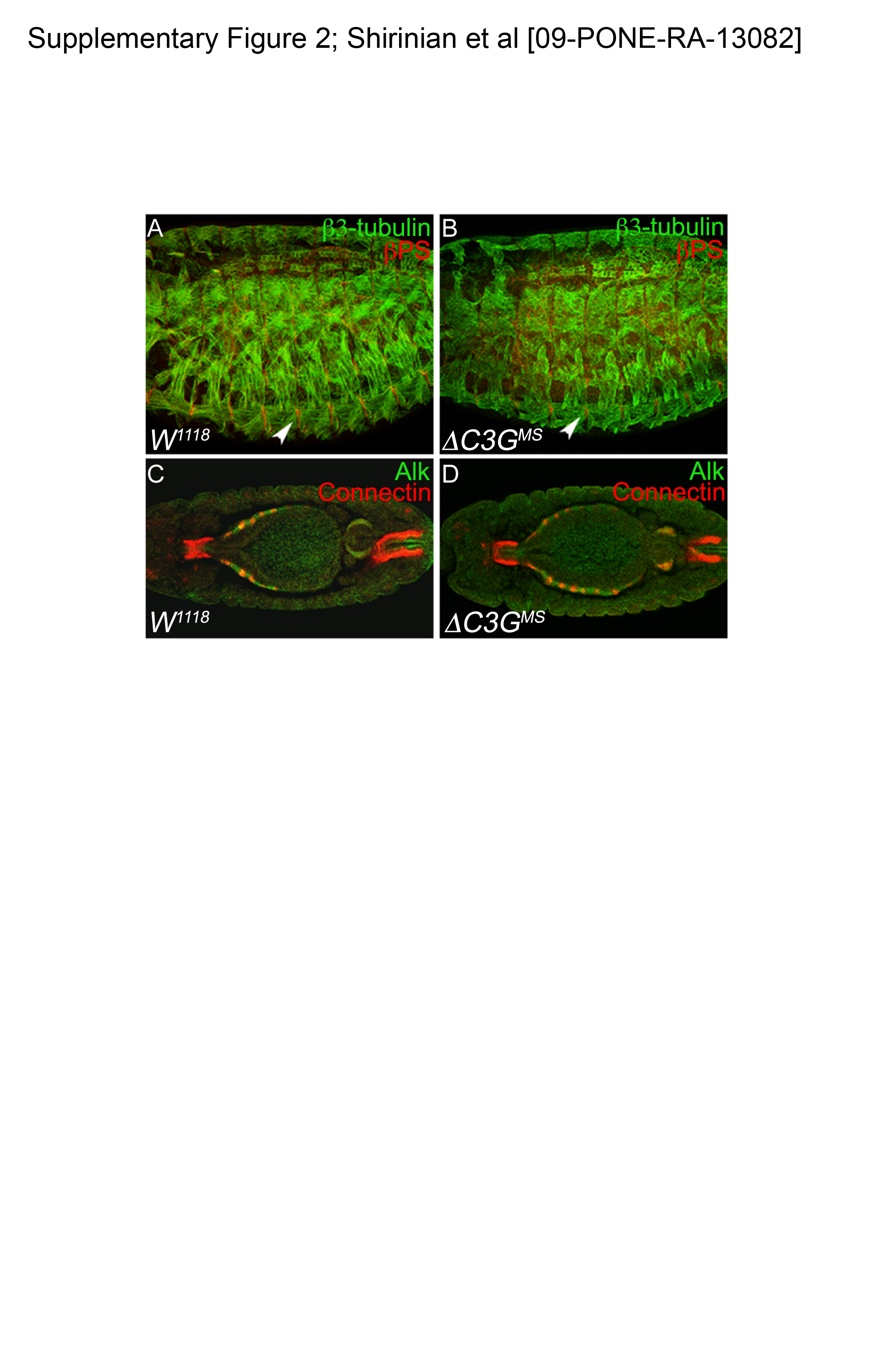

Supplement: Figure S2 — ΔC3GMS mutant embryos undergo normal fusion and migration of somatic and visceral muscles. To visualize the somatic muscle attachment sites, wild type and ΔC3GMS mutant embryos were stained with βPS integrin (red) and β3-tubulin (green) antibodies. βPS integrin localization appears to be normal in ΔC3GMS mutant embryos (A, B). (C, D) Wild type and ΔC3GMS mutant embryos were stained with anti-Alk antibodies (green) to visualize the visceral muscles and anti-Connectin antibodies (red) as a cytoskeletal marker in the visceral mesoderm. The founder cells and fusion competent myoblasts fuse normally in ΔC3GMS mutant embryos (D). (4.02 MB TIF) [file pone.0009403.s002.tif]

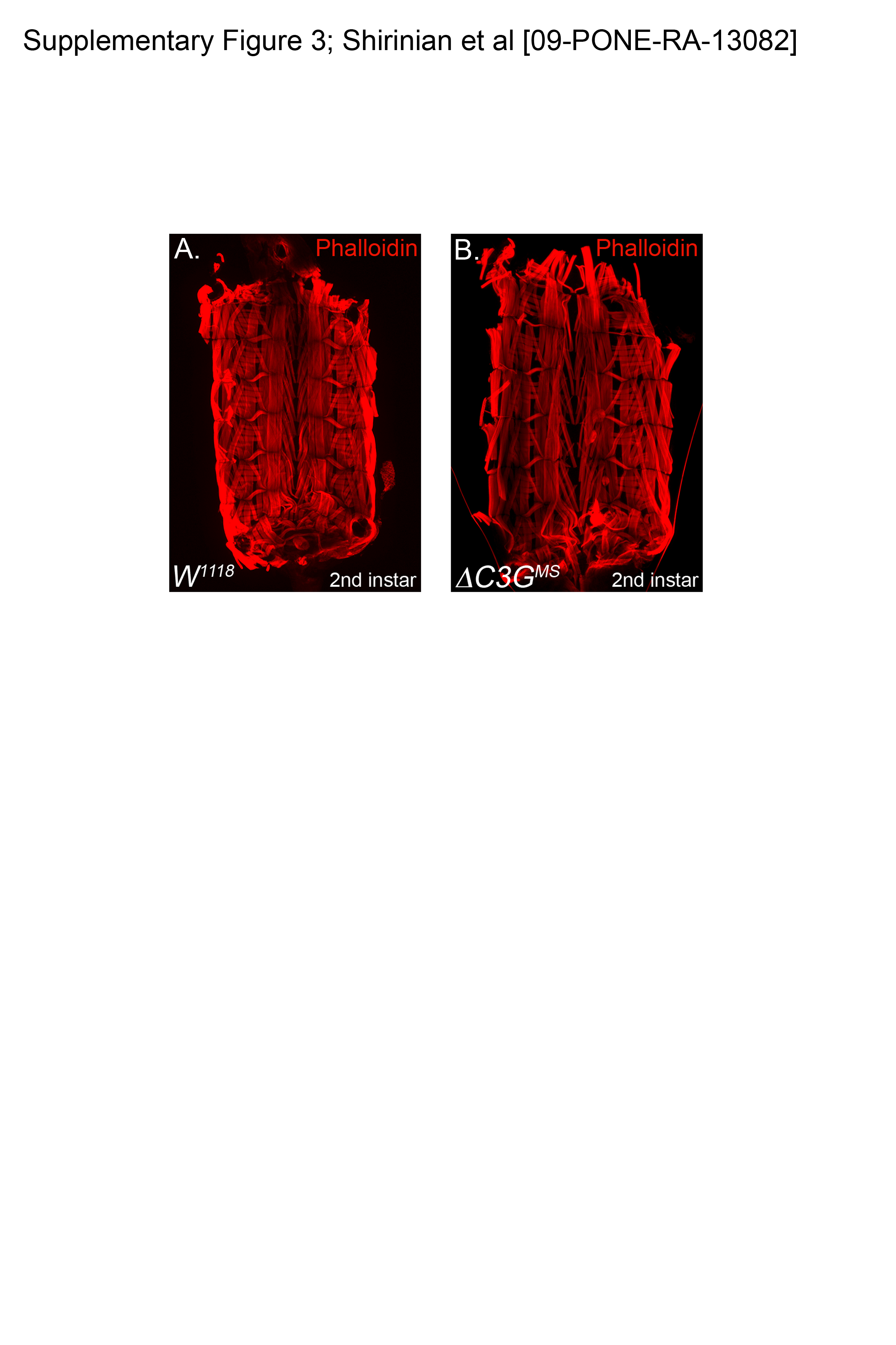

Supplement: Figure S3 — ΔC3GMS mutant larvae display muscle abnormalities already at the 2nd instar stage of development. (A, B) Wild type and ΔC3GMS mutant larvae were dissected at the 2nd instar stage and stained with Phalloidin to visualize the body wall musculature. Whereas wild type animals (A) at this stage display regular and robust longitudinal muscle fibers, the ΔC3GMS mutants (B) are characterized by thin longitudinal muscle fibers that tend to be both mistargeted and in some cases detached (right panel). (2.22 MB TIF) [file pone.0009403.s003.tif]

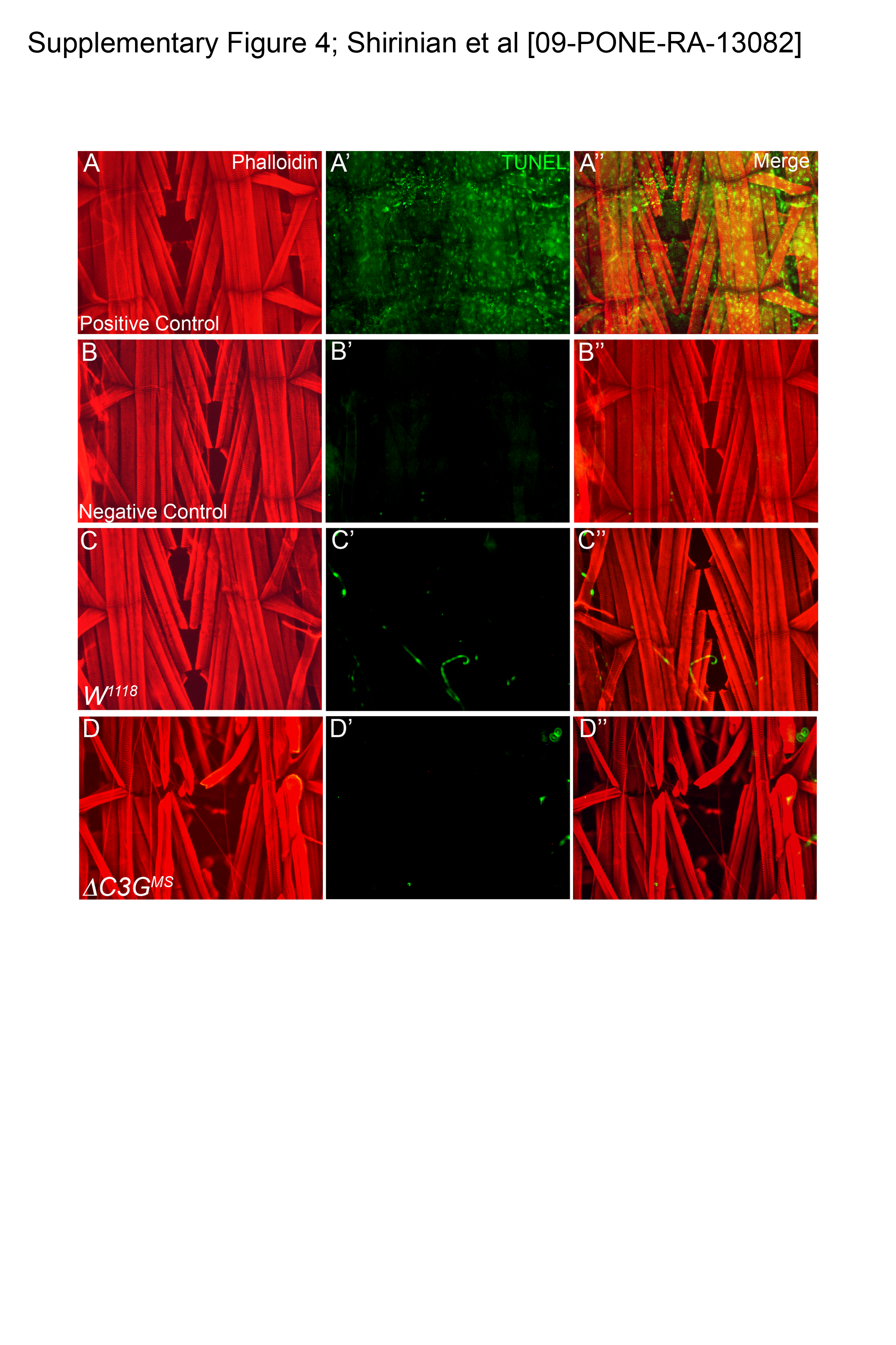

Supplement: Figure S4 — ΔC3GMS mutant muscles do not display increased apoptosis. Third instar wild type and ΔC3GMS mutant larval muscles were dissected and subjected to a TUNEL assay. DNA fragmentation, characteristic for apoptosis, was analyzed by fluorescent labeling (Green). Samples treated with DNAse I were used as positive controls (A'). As negative control samples were treated with the TUNEL reaction mixture, but without addition of the terminal transferase. No apoptosis was observed in ΔC3GMS mutant larva, similar to wild type muscles (D'-C'). (7.43 MB TIF) [file pone.0009403.s004.tif]

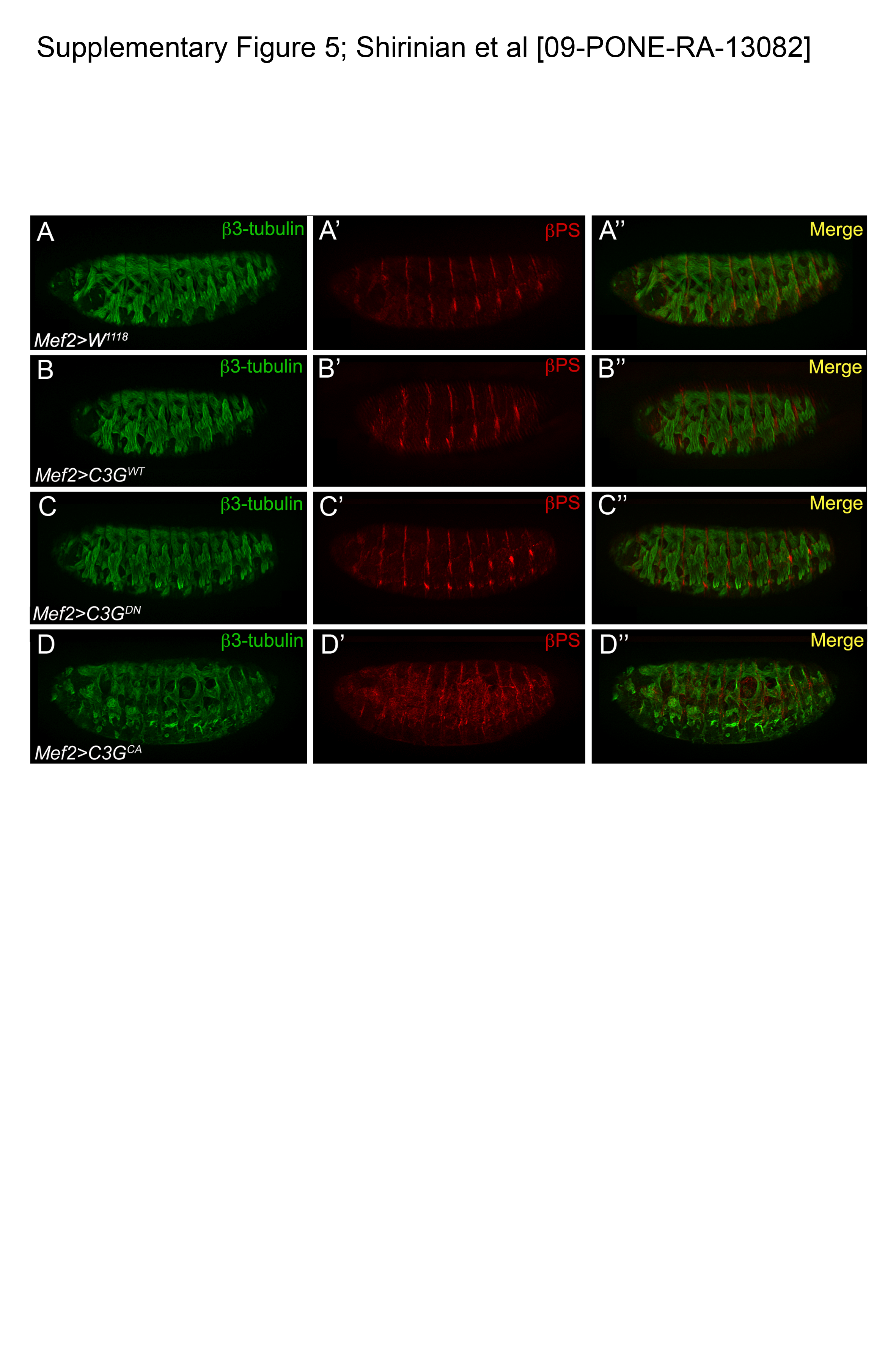

Supplement: Figure S5 — Embryonic muscle development is affected by C3G CA, but not C3G WT or C3G DN misexpression. (A–D) Mef2-GAL4 was used to drive expression of wild type (C3G WT), dominant negative (C3G DN) or activated (C3G CA) specifically in muscles. Late stages embryos were collected and stained with anti-β3-tubulin (green) and anti-βPS (red) antibodies. The integrity of the embryonic somatic musculature and the targeting of integrins to muscle attachment sites was not affected by misexpression of either C3G WT (B) or C3G DN (C). However, misexpression of C3G CA resulted in muscle defects of variable magnitudes (D). (4.03 MB TIF) [file pone.0009403.s005.tif]
